# Loss of ferritin in developing wing cells: Apoptosis and ferroptosis coincide

**DOI:** 10.1371/journal.pgen.1008503

**Published:** 2020-01-02

**Authors:** Anna Karen Hernández-Gallardo, Fanis Missirlis

**Affiliations:** Departamento de Fisiología, Biofísica y Neurociencias, Cinvestav, CDMX, México; German Cancer Research Center (DKFZ), GERMANY

Simone Mumbauer and colleagues have studied ferritin’s role in the context of Hippo signaling within *Drosophila melanogaster* wing primordial cells [1]. Their findings suggest that H and L ferritin subunits may have noninterdependent functions. When subunit H is lost, increased levels of reactive oxygen species and cell death by apoptosis are observed, as well as mitochondrial hallmarks, which may be defined as ferroptosis [2]. Loss of Hippo signaling in this genetic background rescued cell death, despite the reactive oxygen species, which persisted. Does loss of Hippo signaling in a developmental context protect from ferroptosis? In mesenchymal and renal carcinoma cells, however, loss of Hippo signaling correlates with susceptibility to ferroptosis [3, 4]. These apparently contrasting findings call for an explanation.

## Iron and ferritin in development

But first, the basics. No iron, no life. Key protein activities required for living organisms to grow, such as the mitochondrial electron transport chain that supports oxidative phosphorylation or ribonucleotide reductase that generates deoxyribonucleotides for DNA replication, rely on iron cofactors [5]. The iron–ferritin complex is an important regulator of cellular iron bioavailability, storing iron and preventing free iron reactivity [6, 7]. It is therefore unsurprising that, during oogenesis, ferritin iron is transferred to the oocyte. Both maternally contributed ferritin and zygotic expression are required to complete embryogenesis [8]. To investigate whether ferritin also functions in wing development, one unavoidably relies on RNA interference or the formation of mosaic clones through mitotic recombination. Both types of intervention showed that knockdown of the ferritin H subunit, in compartments of the wing imaginal disk, reduces wing size as a result of cell death occurring both cell-autonomously and nonautonomously [1]. These phenotypes were significantly less pronounced following ferritin L subunit knockdown.

## Hippo signaling, wing growth, and oncogenesis

Hippo signaling is required for proliferation arrest and apoptosis in developing imaginal discs [9]. Fisun Hamaratoglu and colleagues in Georg Halder’s laboratory identified the protocadherin Fat and Neurofibromatosis type-2-like homologous proteins, Merlin and Expanded, as upstream regulators of Hippo [10, 11]. More recently, they described how Hippo signaling keeps Ras oncogenic activity in check. Upon loss of Hippo activity, the pathway’s downstream transcription factor Yorkie (otherwise targeted for degradation) induces the expression of Pointed and Capicua, key effectors of Ras signaling [12]. Furthermore, while Ras is active, Yorkie induces a coordinated network of pro-proliferative transcription factors that sustain tumor formation [13]. These studies exemplify how genetics of *Drosophila* wing development inform cancer biology by identifying tumor suppressor mechanisms that limit cell proliferation. The same studies also led to the discovery that the ferritin genes were overexpressed when Hippo signaling was inactivated and to the hypothesis that ferritin induction contributes to the overgrowth phenotype of Hippo loss [1].

## Cell competition, apoptosis, and ferroptosis

During wing growth, the fitter and faster proliferating cells affect a regulated form of cell death on their slower dividing, and presumably less fit, neighbors. This type of cell competition was discovered through generating clones of mutants in ribosomal genes, known as *Minutes* [14]. The strong developmental disadvantage of *Minute* clones compared to wild-type neighbors has proven useful for stabilizing sick neighboring mutant cells that would otherwise be eliminated. Surprisingly, the *Minute* cells outcompeted cells lacking the ferritin H subunit, making the latter an example of “super-loser” cells [1].

Hippo signaling operates to slow down growth and primes cells for apoptosis [15], although cell death is not always prevented when apoptosis is inhibited [10]. Apoptosis inhibitors were not much help to ferritin H mutant cells, in contrast to a genetic manipulation that countered Hippo signaling, which allowed cells to survive [1]. Thus, the question arose whether a different form of regulated cell death was responsible for the elimination of mutant ferritin H cells when Hippo signaling is active in the growing wing tissue.

Enter the new kid on the block: ferroptosis [2, 16]. Three characteristics of this recently described, regulated form of cell death are the following: (1) presence of mitochondrial instead of nuclear fragmentation during the necrotic process, (2) iron induced lipid peroxidation, and (3) inactivation of a dedicated glutathione peroxidase that normally acts as a dominant inhibitor of ferroptosis. Ferritin H knockdown cells showed characteristic mitochondrial defects associated with ferroptosis (ferritin L mutant cells were less affected). Ferritin H knockdown cells also showed signs of oxidative stress, reversed with superoxide dismutase and catalase overexpression [1]. Moreover, in a different study, feeding flies with the ferroptosis-inducing drug erastin caused a reduction in their life span, which was restored by overexpression of the mitochondrial H ferritin subunit [17, 18]. These results, along with the assumption that iron toxicity increases in ferritin H knockdown cells, suggest that ferritin H mutant cells die through the process of ferroptosis. Consistent with this idea, loss of ferritin is also required for erastin-induced ferroptosis in cancer cell lines [19].

It remains to be seen whether lipid peroxidation and *Drosophila* glutathione peroxidase homologs (such as Glutathione peroxidase homolog with thioredoxin peroxidase activity (GTPx-1) [20]) are involved in insect ferroptosis. *Drosophila* hematopoietic progenitors are known to generate reactive oxygen species to differentiate; notably, overexpression of GTPx1 blocked the process of differentiation [21]. Conversely, RNA interference of ferritin H in the same cells (but not of ferritin L) drove massive differentiation [22], pointing to an in vivo setting, myeloid blood cell differentiation in which the ferroptotic pathway may have a physiological role. This observation could be important when considering undesirable effects of ferroptosis-inducing drugs in the context of cancer therapeutics. Further studies are required to establish the genetics of ferroptosis in insects. Newly described players in this cell death pathway could also be rapidly tested [23, 24].

## Ferritin subunits: How and when do they appear to function independently?

**Fig 1:**
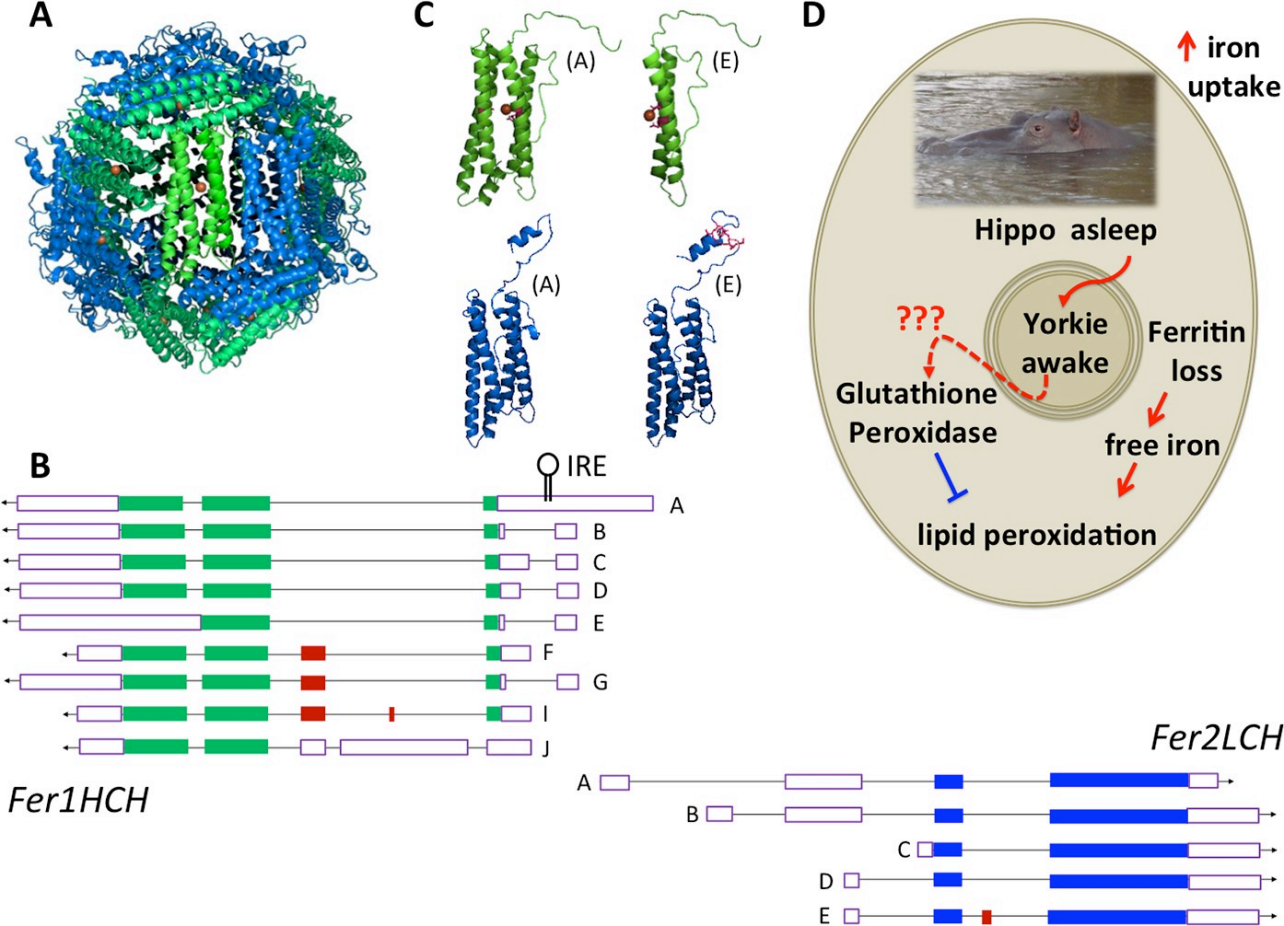
The *Drosophila* ferritin complex and its relationship to ferroptosis. **(**A) Homology model of the assembled protein complex based on PDB: 1Z6O. Ferritin H subunit in green and L subunit in blue. (B) The genes encoding for H and L subunits are linked in a head-to-head orientation. Transcripts are taken from Flybase. ORFs for the ferritin H and L subunits are shown in green and blue, respectively. Red shows alternatively spliced ORFs. The position of an IRE is depicted. Note the H subunit isoform that lacks the leader peptide sequence and is therefore predicted to encode a cytosolic protein (transcript J). (C) Homology model structures for ferritin H subunit monomers resulting from transcripts A and E (green; amino acid side chains coordinating an iron atom at the ferroxidase site are shown) and ferritin L subunit monomers A and E (blue; amino acid side chains are shown for the short, additional N-terminal insertion in isoform E). (D) Simplified scheme showing a *Drosophila* wing cell that has survived ferroptosis or a cancer cell primed for ferroptosis. Iron misregulation is key to both, but whether GTPx-1 is involved in *Drosophila* (like GPx4 is in cancer cells) remains to be seen. Fer1HCH, Ferritin 1 heavy chain homolog; GTPx-1, Glutathione peroxidase homolog with thioredoxin peroxidase activity; IRE, iron-responsive element; ORF, open reading frame; PDB, protein data bank.

The crystal structure of an insect (*Trichoplusia ni*) ferritin strongly influences our thinking that the final product of 12 H and 12 L subunits is the functional ferritin unit [25]. Single particle electron microscopy of *Drosophila* ferritin [26] is consistent with the model structure shown in Fig 1A. Ferroxidase activity required for iron deposition in the ferritin cavity is provided by the H subunit and is essential [6], whereas the L subunits collaboratively generate a binding site for iron, located in the internal cavity of the complex [25], from where iron mineralization is thought to initiate [26]. The embryonic phenotypes of mutant H, mutant L, and a deletion mutant removing both H and L, are very similar [8]. Furthermore, individual knockdown of either H or L expression has been shown to reduce the levels of both subunits [6, 7]. The coupled genomic organization of their respective genes (Fig 1B), which is conserved in insects [27, 28], appears to serve a coregulatory purpose.

Why is it then that interference with ferritin H function in some cells produces stronger phenotypes [1, 22, 29], while the converse is also true (i.e., ferritin L specific phenotypes [30] also exist)? We hypothesize that the reason relates to ferritin assembly regulation [31] as opposed to moonlighting functions for the individual subunits. For example, surrounding cells could provide ferritin L subunits to their neighbors but not vice versa. If true, loss of H chain could equate to loss of ferritin, whereas loss of L chain would have minor effects. Ferritin subunit trafficking between cells has been observed [8], but whether it is the ferritin complexes, the ferritin subunits, or both that traffic remains unknown.

Through the use of different promoters and alternative splicing, nine transcripts encode for five isoforms of the ferritin H subunit, and five transcripts encode for two isoforms of the ferritin L subunit (Fig 1B). The L subunit isoforms differ in their N-termini (Fig 1C), and this is also the case for three H subunit isoforms (Fig 1B). The N-terminus region is where trafficking regulation, such as glycosylation, is expected to act. Intriguingly, the fourth H subunit isoform has an incomplete fold, to which iron binding may still be possible, as three of four ligating amino acids (glutamates 62, 87, and histidine 90) are still present (Fig 1C). Lastly, the fifth H subunit isoform lacks the signal peptide and is therefore predicted to encode a cytosolic ferritin (Fig 1B). The jury is out on how such complexity contributes to cellular and iron homeostasis.

## Is Hippo signaling connected to iron, ferroptosis, and cancer?

The answer seems to be affirmative. In the context of wing development, loss of the ferritin H subunit led to ferroptosis-like death, but additional loss of Hippo signaling reversed this outcome, despite a very high level of reactive oxygen species in these doubly affected cells [1]. Which downstream targets of Yorkie could protect from ferroptosis-like death in this context is unknown (Fig 1D). It is plausible that loss of Hippo signaling directly regulates GTPx-1: Yorkie’s DNA binding partner, Scalloped, binds immediately upstream [12, 32], and Yorkie overexpression led to GTPx-1 overexpression [32], meaning lipid peroxidation could be inhibited as a result (presumably not reactive oxygen species per se).

In contrast, loss of Hippo signaling renders cancer cells susceptible to ferroptosis, but this susceptibility is observed after pharmacological or genetic treatments that ensure the inactivation of glutathione peroxidase [3, 4]. Should loss of Hippo signaling also induce GPx4, then an additional explanation of why active Hippo signaling caused cancer cells to be unresponsive to erastin could be that they had reduced GPx4 to start with. Finally, transferrin receptor induction [3] and ferritin down-regulation in cancer cells undergoing ferroptosis [19] are different ways to increase free intracellular iron. After all, ferroptosis was defined because iron chelators inhibit it [2].

## Conclusion

*Drosophila* genetics offer powerful tools to dissect the molecular mechanisms of ferroptosis and its physiological significance. It appears ferroptosis is evolutionarily conserved.
